# Transcriptomics of morphological color change in polychromatic Midas cichlids

**DOI:** 10.1186/1471-2164-14-171

**Published:** 2013-03-13

**Authors:** Frederico Henning, Julia C Jones, Paolo Franchini, Axel Meyer

**Affiliations:** 1 Laboratory of Zoology and Evolutionary Biology, Department of Biology, University of Konstanz, Universitätsstraße 10, Konstanz 78457, Germany; 2 Zukunftskolleg, University of Konstanz, Konstanz, Germany

**Keywords:** RNAseq, Color change, Melanophore, Differential expression, Tyrosinase genes, Midas cichlids

## Abstract

**Background:**

Animal pigmentation has received much attention in evolutionary biology research due to its strong implications for adaptation and speciation. However, apart from a few cases the genetic changes associated with these evolutionary processes remain largely unknown. The Midas cichlid fish from Central America are an ideal model system for investigating pigmentation traits that may also play a role in speciation. Most Midas cichlids maintain their melanophores and exhibit a grayish (normal) color pattern throughout their lives. A minority of individuals, however, undergo color change and exhibit a distinctive gold or even white coloration in adulthood. The ontogenetic color change in the Midas cichlids may also shed light on the molecular mechanisms underlying pigmentation disorders in humans.

**Results:**

Here we use next-generation sequencing (Illumina) RNAseq analyses to compare skin transcriptome-wide expression levels in three distinct stages of color transformation in Midas cichlids. cDNA libraries of scale tissue, for six biological replicates of each group, were generated and sequenced using Illumina technology. Using a combination of three differential expression (DE) analyses we identified 46 candidate genes that showed DE between the color morphs. We find evidence for two key DE patterns: a) genes involved in melanosomal pathways are up-regulated in normally pigmented fish; and b) immediate early and inflammatory response genes were up-regulated in transitional fish, a response that parallels some human skin disorders such as melanoma formation and psoriasis. One of the DE genes segregates with the gold phenotype in a genetic cross and might be associated with incipient speciation in this highly “species-rich” lineage of cichlids.

**Conclusions:**

Using transcriptomic analyses we successfully identified key expression differences between different color morphs of Midas cichlid fish. These differentially expressed genes have important implications for our understanding of the molecular mechanisms underlying speciation in this lineage of extremely young species since they mate strongly assortatively, and new species may arise by sexual selection due to this color polymorphism. Some of the human orthologues of the genes identified here may also be involved in pigmentation differences and diseases and therefore provide genetic markers for the detection of human pigmentation disorders.

## Background

**Figure 1:**
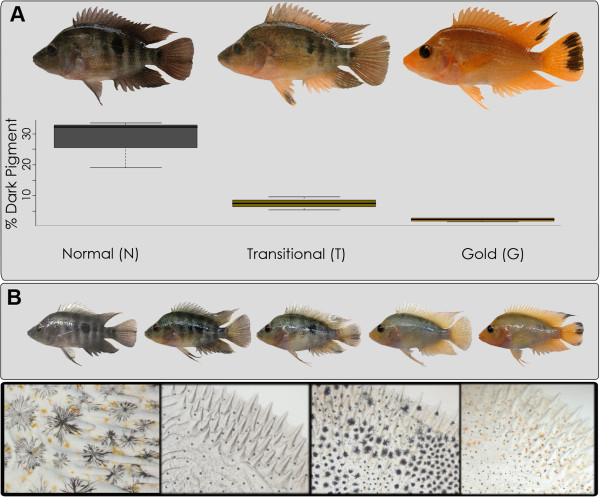
**Morphological color change in the Midas cichlid.** A representative individual from each of the three groups (N, T and G) is shown in **A**. The groups are ordered along the X axis according to the melanophore density. The percentage of the scale area that is dark is shown in the box-and-whiskers plot. Plotted values represent the median (bar) and interquartile range (box) for the percentage of scale area covered by dark pigment in Normal, Transitional and Gold fish (n = 4 for each group). An ontogenetic series of a single individual is shown in **B**. Viable melanophores in dispersed and aggregated states are shown in **C** and **D**, respectively. Melanophores of transitional fish showing a mixture of aggregated, dispersed and dead melanophores are shown in **E**. Scale of a gold fish completely lacking melanophores (**F**). (**B**). A decrease in the number of melanophores can be readily noted both from the total amount of dark pigment in the body (**A**) or individual scales (**D-F**).

Animal pigmentation is a tractable and relevant trait for understanding key issues in evolutionary biology such as adaptation, speciation and the maintenance of balanced polymorphisms. While these evolutionary processes themselves have attracted much research attention, apart from a few cases [1-7] the genetic changes associated with speciation and adaptation remain largely unknown. In addition to its evolutionary and ecological relevance, pigmentation genetics also has medical implications [8], since, due to evolutionary conservation of the main pigmentation pathways, many pigmentation genes in fish and other animal models have contributed to our understanding of human pigmentation disorders [9-12] and of phenotypic differences and human evolutionary history [13-15]. In the present paper, we investigate gene expression differences that correlate with the ontogenetic development of a color trait thought to underlie speciation-with-gene flow in Neotropical cichlids (Figure 1A).

Cichlid fishes in general are well known for their rapid radiation and speciation [16]. Morphological diversification, including a diverse range of color traits, are particularly striking among the haplochromine cichlids in Lakes Victoria and Malawi in eastern Africa which, with several hundred endemic species each, make up the largest recent adaptive radiation of vertebrates [17-20]. The Midas cichlid species complex (*Amphilophus*) from the lakes of Nicaragua also constitutes a model system for the study of speciation and radiation by providing one of the very few accepted empirical examples of sympatric speciation [21,22]. Two of the main processes thought to be driving divergence in these fish are ecological disruptive selection and color-based assortative mating [21,23-25]. Several populations of Midas cichlids are characterized by a conspicuous color polymorphism. In many of the species in this complex there are two color morphs: “normal” and “gold”. The normal morph is always much more common (hence the term “normal”) and these individuals show a grey-black coloration. The gold morph shows a bright orange or gold color (Figure 1A) and gave these cichlid fishes their common name, the “Midas” cichlids, in reference to the Greek god Midas who turned everything that he touched into gold [25]. Some individuals lose melanophores, purportedly via melanophore cell death, and become gold while others remain normal in color throughout their lives [26] (Figure 1A). Color change does not progress uniformly (Figure 1B) and occasionally dark regions can persist even years after color change. The inheritance patterns suggest that this polychromatism is determined by a single locus, with the gold allele dominant to the normal one [27,28]. The timing of the onset of this color change is variable and can begin when an individual is only a few months old, but can also, in a few individuals happen at a much older age - up to several years [25,26].

Based on observations [29] and experiments from their natural habitats as well as the laboratory, these different morphs are known to mate assortatively [25] and significant genetic differentiation between morphs has been found in two crater lakes [21,23,24]. Thus, suggesting that this color trait with a simple, mendelian genetic architecture underlies some of the speciation processes in the Midas cichlids. This simple genetic basis can also contribute to understanding the genetic architectures that are more permissive of sympatric speciation in general. Linkage or pleiotropy of the mate choice cue and preference loci is expected to exist in this situation [30]. Although this was traditionally considered rare, a few empirical examples have recently been published [31-34].

Among teleosts pigmentation has been associated with mate recognition, mate choice, shoaling behavior, camouflage and warning coloration and premating isolation during speciation [12]. In vertebrates more generally, mouse coat color and the array of color patterns among zebrafish (*Danio rerio*) and its relatives, are some of the most useful and well-explored systems for identifying the molecular mechanisms of color change and color pattern development ([12], reviewed in [35]).

Melanophores are chromatophores, or color bearing cells, that are derived from the neural crest and are responsible for production and storage of melanin in specialized vesicles (melanosomes) [36]. Chromatophores are cells specialized in the storage and/or synthesis of light absorbing (true) pigments or light reflecting structures [37]. Mammals and birds exhibit only a single pigment cell, the melanocyte (called melanophores in poikilothermic animals) that produces two types of pigment: eumelanin, responsible for black to brown color; and pheomelanin, responsible for red to yellow color [38]. In contrast, six kinds of chromatophores are known in poikilothermic vertebrates: melanophores (black or brown), xanthophores (ochre or yellow), erythrophores (red), leucophores (whitish), iridophores (metallic or iridescent) and cyanophores (blue) [39].

In teleost fish both morphological and physiological processes can play a role in coloration. *Morphological* skin color change in fish is becoming increasingly recognized as a more broadly applicable phenomenon brought about by a large variety of factors [37]. This type of color change is defined as occurring via variations in skin pigment concentrations and in the morphology, density and distribution of chromatophores in the three-dimensional organization of the integument – such color changes are relatively slow, occurring over days or weeks [37]. Changes in the external coloration of teleosts are also influenced by *physiological* phenomena. Primary physiological color changes are caused by the direct effect of environmental factors, such as light, on pigment migration [37], and secondary physiological color changes are those where pigment translocation is mediated by the nervous and endocrine systems. The latter mode of color change involves a number of different factors including adrenocorticotropic hormone (ACTH) and alpha-melanophore-stimulating hormone (-MSH). Color responses controlled by the nervous system have an almost instantaneous effect, and those color changes mediated by the endocrine system tend to happen within minutes or hours [39].

Specifically, the molecular mechanisms behind the loss of melanophores are largely unknown. The coloration patterns that are most closely aligned to that seen in the Midas cichlids is perhaps the orange-blotch (OB) phenotype in the cichlid fish of Lake Malawi, East Africa [3]. Interestingly, the OB phenotype, caused by a cis-regulatory mutation in the PAX7 gene, might increase camouflage in females but, at the same time, disrupts species-specific male color patterns used for mate recognition. This sexual conflict is thought to have been resolved by linkage to a novel female sex determining locus [3]. The synthesis of black melanin (or eumelanin) in vertebrates more generally, involves the members of the tyrosinase gene family and melanosomal transporters [40]. Interestingly, the late onset of melanophore cell death in Midas cichlids may also be caused by similar mechanisms to human pigmentation disorders, such as white hairs and vitiligo, whose genetic architectures are also still largely unresolved [41].

The aim of the present study is to identify differences in gene expression associated with the transition from normal to gold coloration in Midas cichlid fish. We use next-generation sequencing to uncover genes that underlie morphological color change in this system. In the present study, we consider *color-change genes*, as genes whose expression patterns correlate with the process of transition from normal to gold. The correlation of their expression with the process of color change suggests that they are molecularly (and perhaps causally) related to the transition from normal to gold although these are not necessarily expressed in pigment cells and/or directly related to pigment production.

**Figure 2:**
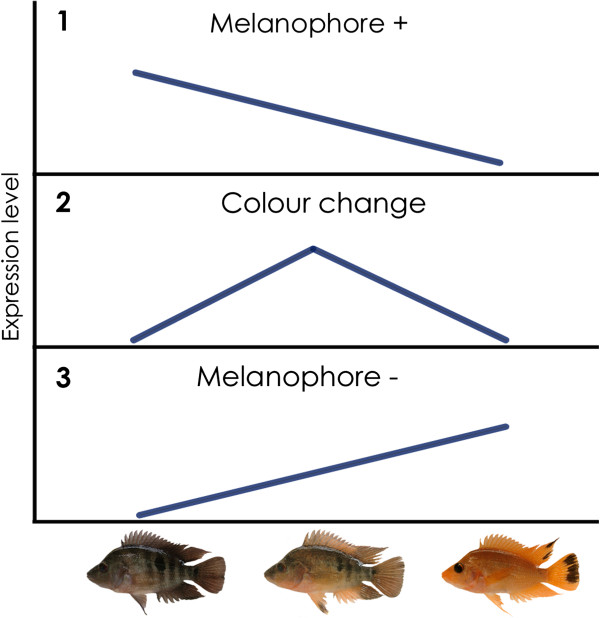
**Expected differential expression patterns.** Genes expressed by melanophores are expected to correlate positively with the number of melanophores present (**1**). Likewise, genes under negative regulation should correlate negatively with the amount of melanophores (**3**). Expression pattern **2** can be anticipated for genes involved in the process of color change.

Melanophore density decreases during transition (Figure 1A): N (high), T (intermediate) and G (low or absent altogether). Because of this natural progression, three different patterns of DE are expected (Figure 2). DE pattern 2 (up-regulation in T) represents genes related to the process of morphological color change. DE pattern 1 (*Melanophore +*) is expected for genes that are expressed specifically by melanophores. In this case, expression level is expected to correlate with the number of melanophores and decrease from N to G. DE pattern 3 (*Melanophore –*) is expected for those genes that are under negative regulation of melanophores. Accordance of well-known melanogenesis genes with pattern 1 provides internal validation for the experiment.

Here we analyzed genome wide expression levels in three contrasting phenotypes that represent distinct stages of color transformation in these fish. Normal, transitional and gold phenotypes were studied in mature, age matched, sibling fish (Figure 1) using six biological replicates of each phenotype, and two technical replicates of each library. We identify both well-known and uncharacterized genes associated with melanophore maintenance, cell death and clearance as well as potential regulatory target genes. Our results illustrate the conservation of genes affecting pigmentation and suggest that some of our DEGs are potential markers for human pigmentation disorders. Interestingly, one of the genes that we found to be upregulated during transition also segregates with the gold phenotype in a genetic cross and is possibly associated with incipient speciation in this system.

## Results and discussion

The Midas cichlid fish system provides a rare example of sympatric speciation, where color-based assortative mating is one of the processes thought to be driving divergence in these fish [21,23,24]. Here, using transcriptome wide next generation sequencing technology (Illumina) we identified key expression differences between different pigment morphs (normal, N; transitional, T; gold, G) in the same Midas cichlid species (Figure 1). Some of the differentially expressed genes detected have also been implicated in parallel pigmentation traits in other organisms and in human pigmentation disorders [13,42-46].

**Figure 3:**
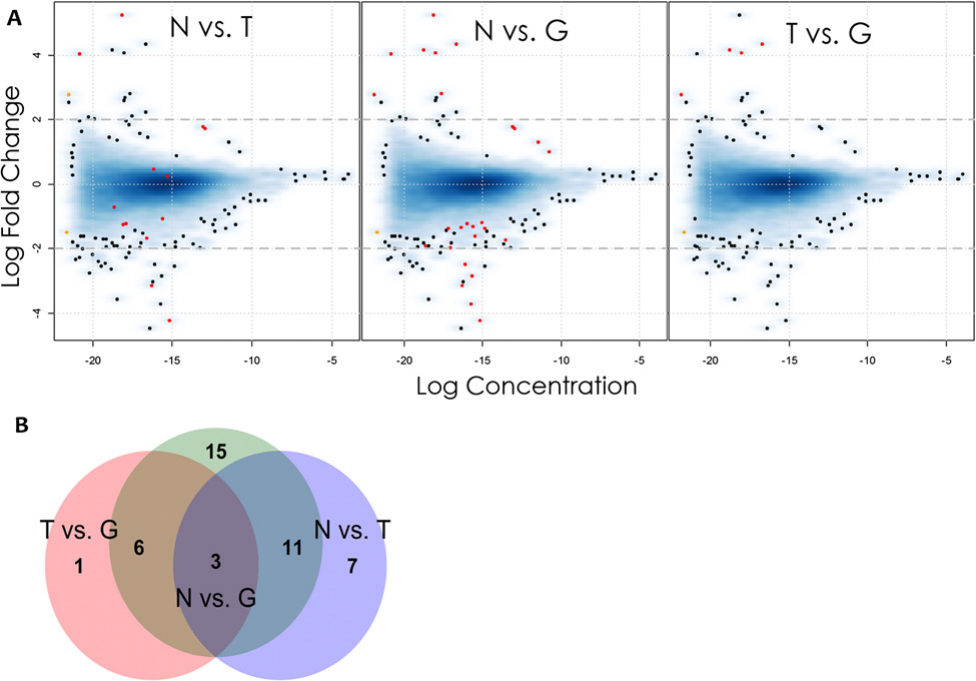
**RNAseq ‘MA’ plot.** (**A**) For each of the three comparisons genes found to be DE using edgeR comparisons are shown in red. Venn diagram showing the overlap of DEG among the three comparisons (**B**).

We sampled the scale tissue from 18 full-sib Midas cichlids originating from heterozygous gold parents (n = 6, per group) for the RNAseq experiment (see also Methods). Sequencing generated 264,402,166 raw reads and this number was reduced to 191,454,241 sequences after the cleaning pipeline was implemented (see Additional file 1: Table S1 for details). The latter sequences were used to build the de novo assembly. Velvet/Oases produced a high number of transcripts that were clustered by CD-HIT-EST into 299,124 contigs. The following approaches based on the BLASTx algorithm and MySQL queries (see Methods section for details) were applied in order to restrict the analyses to annotated sequences and resulted in the final de novo assembly of 20,420 sequences. Contigs shorter than 200 bp were discarded from further analyses since they could contain artefacts derived from cDNA synthesis, sequencing and contamination. The contigs ranged in size between 200 bp (chosen threshold) and 17,407 bp (average length of 3,066 bp and a N50 value of 4,168 bp). The average nucleotide-wide coverage was estimated to be 188.3x. An average of 8.5 M reads from each library (Additional file 2: Figure S1) were mapped to the Midas *de novo* assembly and to the 21,461 tilapia reference sequences. The dispersion and concentration of fold change values are displayed in Figure 3A.

### Patterns of differential gene expression in color change

Three separate analyses of differential gene expression were carried out using the edgeR [47], DESeq [48] and baySeq [49] R packages (R v. 2.14.02) (Table 1). A total of 44 differentially expressed genes (DEGs) (Table 2) were identified in at least one analysis method (Figure 2 and Table 2).

**Table 1:** Analysis methods employed for differential expression detection in this study

**Analysis**	**Package**	**Comparison**	**Assembly**
a	edgeR	Pairwise	Midas cichlid
b	DESeq	Pairwise	Midas cichlid
c	baySeq	Pairwise	Midas cichlid
d	baySeq	Multiple	Midas cichlid
e	edgeR	Pairwise	Tilapia
f	DESeq	Pairwise	Tilapia
g	baySeq	Pairwise	Tilapia
h	baySeq	Multiple	Tilapia

**Table 2:** All differentially expressed genes (DEGs) detected in this study

	**Annotation**	**Analyses**	**Comparison**	**Expression pattern**	**Best hit**
1	TYR	g	N vs. G	1	XP_003441635.1
		c	T vs. G		
2	TYRP1a	a,b,c,d	N vs. G	1	XP_003450374.1
		a,c,d	N vs. T		
3	TYRP1b	c,e,f,g,h	N vs. G	1	AAL84110.1
		d	N vs. T		
		d,e,h	T vs. G		
4	SLC24a5	a,c,d,g,h	N vs. G	1	Q49SH1.1
		a,c,g,h	N vs. T		
5	PMELa	a,b,c,d,e,g,h	N vs. G	1	AAI29134.1|
		d	N vs. T		
		a,b,c,d,e,f,h	T vs. G		
6	MREG	a,b,c,d,e,fg	N vs. G	1	NP_001002167.1
		d	N vs. T		
7	CXCL13/IL8	b,c,d	N vs. G	2	XP_003444508.1
		a,c,d	N vs. T		
8	CX41.8	e	N vs. G		AAI63086.1
9	SLC6a15	c,d,g,h	N vs. G	2	XP_003449204.1
		a,c,d,g,h	N vs. T		
10	Uncharacterized	a,b,c,d	N vs. G	-	NP_001091866.1
		a,d	N vs. T		
11	Uncharacterized	a,c,d	N vs. G	2	XP_003460123.1
		a,c,d	N vs. T		
12	RT 1	a,c,d	N vs. G	1	NP_001025268.1
		a,c,d	N vs. T		
		b	T vs. G		
13	Uncharacterized	e.h	N vs. G	3	BAE33391.1|
		h	N vs. T		
14	Uncharacterized	g,h	N vs. G	2	XP_003460121.1
		g,h	N vs. T		
15	RIMKA	c,d	N vs. G	2	XP_003444930.1
		c,d	N vs. T		
16	RT 2	a,c	N vs. T	1	BAC82613.1
17	Uncharacterized	g	N vs. T	1	-
18	FYNa	g	N vs. G		NP_001007287.1
19	Uncharacterized	e	N vs. G	1	XP_002663842.1|
20	PKHD1L1	h	N vs. G	-	XP_003447322.1
		h	N vs. T		
21	TRIM16	e	N vs. G	3	XP_001921089.3|
22	Uncharacterized	g	N vs. T	2	XP_003460506.1
23	Uncharacterized	c	T vs. G		XP_003455740.1
24	RASEF	a	N vs. G	3	XP_003446171.1
25	BDH1-like	a	N vs. G	3	XP_003453238.1
26	TTN-like	d	N vs. G	3	A2ASSVI.1
		d	N vs. T		
27	PTGIS	a	N vs. T	2	XP_003441326.1
28	IER2	a	N vs. G	2	XP_003454129.1
29	Uncharacterized	c	N vs. T	-	XP_003199799.1
30	Uncharacterized	c,d	N vs. G	-	XP_003440066.1
		c,d	N vs. T		
31	Uncharacterized	a	N vs. G		EAW84958.1|
32	CRFB4	a,c	N vs. G	3	XP_003443307
33	Uncharacterized	a	N vs. G		BAE33391
34	ZBTB20	a	N vs. G	3	XP_003446897.1
35	CNFN	a	N vs. T	2	XP_003457068.1
36	FAM89a	d	N vs. T	1	XP_003456674.1
		d	N vs. G		
37	STC1	a,c	N vs. T	1	XP_003452723.1
38	C-FOS	a	N vs. G	2	P53450.1
39	MC6AST5	a	N vs. T	1	XP_003447395.1
40	WFDC2	d	N vs. G	1	XP_684531.2
		d	N vs. T		
41	CCDC9	a	N vs. G	3	XP_003456447.1
42	RTN3	c	N vs. T	1	XP_003459827.1
43	RT-TF2	a	T vs. G	1	AAC33526.2
44	JUNb	a	N vs. G	2	XP_003443124.1

Letters indicate under which analysis method (see Table 1) DE was detected.

The partial, rather than total, overlap between the results obtained with the different analysis packages is likely related to the different methods used to estimate dispersion and to calculate normalization factors [47-49]. A frequent solution to avoid false-positives is to only consider the results that overlap in all analyses. However, this approach is conservative (i.e. propagates false-negatives from a single analysis) and is not suitable for studies aiming at identifying a large number of candidates for subsequent analysis. The present study illustrates this quite well in the case of genes such as TYRP1, SLC24a5 and TYR. These are well-recognized melanophore-markers and, as expected, conformed to expression pattern 1. Nevertheless none of these genes were identified by all analysis methods and all three were probably false negatives in the analyses using DESeq. The expression of housekeeping genes does vary, but does not show obvious trends or significant differences across groups (Additional file 3: Figure S2).

**Figure 4:**
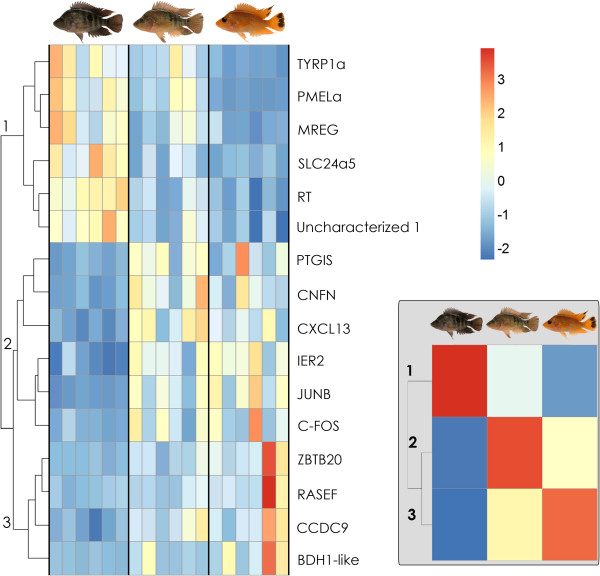
**Heatmap.** Grouping of the top DEG from analysis a (Table 1) based on their relative expression intensity (see Methods section). Numbers indicate DE patterns shown in Figure 2. Results of clustering with *k* = 3 are shown at the top right.

The overlap between the three different comparisons (N *vs.* T, N *vs.* G and T *vs.* G) is shown in Figure 3B. The three expression patterns outlined in Figure 2 can be identified by clustering analysis and are shown in Figure 4. Pattern 1 (positive correlation with melanophore density) is found mainly in well-known pigmentation genes, providing internal validation. Genes more clearly up-regulated during transition (candidate color change genes) were those involved in inflammatory and stress response.

### Candidate color-change genes

DEGs that were up-regulated in T (and in some cases also in G) involved immediate-early genes related to cellular response to stress (IER2, JUNb, C-FOS) and recruitment of B cells (CXCL13). The CXC13 chemokine is similar in sequence to the gene described [50] in the Japanese flounder (*Paralichthys olivaceus*) where it was found to have a function in immune and inflammatory responses. JUNb and C-FOS are known targets of *microphthalmia associated transcription factor* (MITF) regulation [51]. Interestingly, the up regulation of the transcription factor JUNb and C-FOS, as seen here, was previously implicated in the transformation of melanocytes to melanomas in humans [43]. Cornifelin (CNFN) was also upregulated in T. A similar expression pattern of upregulation of CNFN was detected in psoriatic skin [52,53]. Psoriasis is an inflammatory, immune disease in humans characterized by the infiltration of chemokines to the skin tissue [42].

We note that an uncharacterized gene, which includes a lysozyme domain, maps to the *gold* candidate region as defined by positional cloning (S. Fukamachi, F. Henning and A. Meyer, work in progress, see also [28]). Microsatellite and SNP markers located in the vicinity of this gene segregate with the *gold* phenotype in a F_2_ mapping panel confirming linkage. This result raises the exciting possibiity that this uncharacterized gene is the causal gene underlying the gold phenotype. This hypothesis is currently under investigation and will be addressed in future work.

The other color genes analyzed (MC1R, CSF1Ra, KIT, KITLG, SLC45a2) did not conform to any expected pattern or show clear trends (Additional file 4: Figure S3). Although some of these genes clearly showed more variation and higher expression values in the T and G groups (MC1R, CSF1Ra) as reported previously [28].

### Conservation of color genes

Expression pattern 1 (Figure 2) corresponds predominantly to genes related to melanosomal pathways (http://www.espcr.org/micemut/). These were, for example, the tyrosinase gene family members (TYR, TYRP1, DCT), pre-melanosomal protein a (PMELa, also referred to as silva), melanoregulin (MREG), sodium calcium transporter 24a5 (SLC24a5), and in addition one uncharacterized and one reverse-transcribable element. We find that expression pattern 1 is shown in genes expressed by melanophores and related to melanosome components and differentiation [40]. This pattern is expected given the decreasing melanophore densities of normal to transitional to gold fish, and provides internal validation for the present experiment. The expression of these genes was also found to be higher in dark bars relative to light bars in a recent RNAseq study in stickleback [54].

We show that parallel phenotypes can evolve in different lineages by using genes of the same pathways. The zebrafish *golden* gene (slc2415) for instance, was found to be differentially expressed across the Midas cichlid color morphs. Given the differences between the zebrafish and Midas golden phenotypes (constitutive vs. late onset) and the lack of detected sequence differences in the latter, it appears that the expression differences in this gene are downstream consequences of melanophore death in the Midas, which contrasts with its upstream role in causally determining the phenotype in zebrafish [13]. Since some of these genes underlie similar phenotypes in other organisms [13,44], it would appear that they could potentially be good candidates for the Midas cichlid *gold* locus. However, none of these genes map to the gold locus as determined by positional cloning experiments (S. Fukamachi, F. Henning and A. Meyer, work in progress, see also [28]). Nevertheless, the coding regions of the genes known to underlie similar phenotypes in other organisms were sequenced from gold and normal samples to rule out an obvious difference that could account for the phenotype. But, so far no potentially causal polymorphisms (missense or nonsense) were detected.

### Tyrosinase gene family

**Figure 5:**
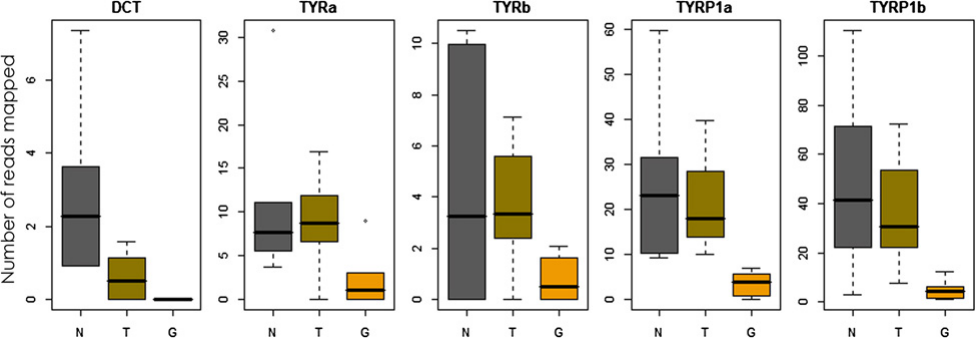
**Expression levels of members of the tyrosinase gene family in three different Midas cichlid fish color morphs.** The number of mapped reads was normalized using the method implemented in edgeR.

The five members of the tyrosinase gene family (DCT, TYRa and b, TYRP1a and b) were clearly negatively correlated with the amount of melanophores in the tissues of the different groups (Figure 5). This is consistent with the role of this gene family in the enzymatic conversion of tyrosine to melanin. TYR is the rate-limiting enzyme in the cascade that produces melanin [55]. DCT is expressed exclusively by melanophores and melanoblasts (melanophore precursors). The level of expression of this gene was found to be very low, with a maximum number of 8 mapped reads in one normal sample. This naturally precludes it from being detected in the global DE analyses.

Despite having retained functional paralogs of several pigmentation pattern genes, the expression patterns of the paralogs seem redundant. This redundancy is consistent with the observed pattern in other teleosts. The impact of the FSGD on the evolution of coloration was recently reviewed [55-57]. In particular, the TYRP1 paralogs were found to have functionally diverged in a lineage-specific manner: TYRP1a is expressed in melanophores and TYRP1b in the retinal pigment epithelium in medaka. The expression of both paralogs overlaps in zebrafish [56]. Here, one copy of MREG and PMEL was found to be DE and this might also indicate that they diverged in function from their paralogs.

## Conclusions

RNAseq was successfully employed to investigate the transcriptome wide expression levels in a non-model organism, the Midas cichlids of Nicaragua. The different color morphs examined here are known to mate assortatively and have diverged significantly suggesting that genes differentially expressed in the different morphs may be playing a role in speciation. We identified both well-known and as of now uncharacterized genes associated with melanophore maintenance, cell death and clearance as well as potential regulatory target genes (Table 2, Figure 4). Our results also illustrate how parallel phenotypes can evolve in different lineages by using genes of the same pathways (e.g. tyrosinase gene family, SLC24A5, PMEL). Other genes that are known to determine similar phenotypes in other organisms [3,4,14,15,58,59] did not show clear trends in our study. The DEGs detected here likely represent novel pigmentation genes of interest to evolutionary biologists generally and to the community of cichlid biologists in particular as candidates for influencing naturally occurring and also parallel phenotypes [60,61]. Therefore, further characterization of the unknown DEGs identified in the present study is warranted. Interestingly, DEGs found to be upregulated in transitional fish also bear resemblance to patterns observed in melanoma formation [43] and psoriasis [52,53]. The evolutionary conservation of the major pigmentation pathways [55] opens the possibility that the other genes that conformed to expression pattern 2 (Figure 4) also constitute potential markers for the development of melanoma or other pigmentation disorders.

## Methods

### Samples

We used full-sib Midas cichlids originating from heterozygous gold parents of a laboratory strain of *Amphilophus citrinellus* segregating for the gold polymorphism. Approximately 200 juveniles were kept for nine months in 2011 in a 500 l freshwater tank under standard light/dark conditions (12:12), at 26°C in the Animal Research Facility at the University of Konstanz, Germany. Under these conditions, color change starts to occur at around 7 months of age (independent of body size). However, a period of nine months was necessary to ensure the presence of sufficient samples of each of the three groups of fish analyzed (N, T and G) while keeping the age difference at a minimum. We chose fish that were clear representatives of the three different color stages (N = fish showing no signs of gold coloring, T = fish with clear patches of both gold and normal coloring throughout the body, G = complete, or almost complete gold coloring throughout the body).

A total of 10–15 scales were collected from both flanks posterior to the pelvic fins and kept in RNA Later at 4°C overnight. The RNA later was removed prior to homogenization. Five-eight scales (depending on the average size) and 1 ml Trizol (Invitrogen, Carlsbad, CA, USA) were homogenized in 1.5 ml tubes using the Tissue Lyser (Qiagen, Valencia, CA, USA). RNA was extracted according to the manufacturer’s recommendations, treated with DNAse I and purified in spin columns (Qiagen). Quantification and integrity was assessed using a Ribogreen assay and a Bioanalyzer 2100 (Agilent Technologies, Waldbronn, Germany). All samples had RNA integrity values above 8.0.

### Illumina sequencing

Libraries were generated using the Illumina TruSeq RNA sample preparation kit (Low-Throughput protocol) according to the manufacturer’s instructions (Illumina, San Diego, CA). Briefly, 1 ug of RNA was subjected to mRNA selection using poly-T oligo-attached magnetic beads followed by chemical fragmentation (6 min, 94°C). The cleaved RNA fragments were then copied into first strand cDNA using SuperScript II reverse transcriptase (Invitrogen) and Illumina proprietary random hexamer primers. After second strand synthesis using Illumina-supplied consumables, the cDNA was amplified with reagents of the same kit according to the manufacturer’s protocol and ligated to barcoded adapters. The final libraries were amplified using 15 PCR cycles. Library quantification and quality assessment was performed on a Bioanalyzer 2100. Equimolar-pooled samples were loaded in four lanes of the Illumina flowcell. Technical replicates were obtained by loading each library in two different lanes. Paired-end sequencing of clustered template DNA on the Genome Analyzer IIx was performed using four-color DNA Sequencing-By-Synthesis (SBS) technology with 151 cycles (72 cycles for each paired-read and seven cycles for the barcode sequences).

### Quality assessment, assembly and read mapping

We used SeqPrep (https://github.com/jstjohn/SeqPrep) to remove any reads that were contaminated with an Illumina adapter. SeqPrep was also used to merge overlapping paired-end reads into single longer reads. To avoid the generation of incorrect sequences, the minimum overlapping length was set to 15 bp, and one mismatch was allowed only when the overlapping region was ≥ 50 bp. Next, the resulting sets of paired- and single- (merged) reads were filtered for quality using CLC Genomics Workbench v4.9 (CLC bio, Aarhus, Denmark). The trimming module of CLC was used to remove the first 13 bp from the 5^′^ end of all reads due to ambiguous GC content. The latter is a common bias in Illumina transcriptome sequencing caused by random hexamer priming [62]. Low quality reads (CLC “limit” set to 0.02) and reads shorter than 20 bp were discarded. Finally, bacterial, fungal, viral and protozoan reads were removed by aligning each Illumina library to all sequenced genomes of these organisms (source: NCBI Reference Sequence, RefSeq, March 2012) using Bowtie v2.0.0-beta5 [63].

In the present study, besides building a de novo assembly, we also took advantage of the Tilapia transcriptome assembly constructed by the Broad Institute. The construction of a de novo assembly is a challenging aspect of work on non-model organisms because considerable resources must be allocated to produce an assembly that reliably represents the complete transcriptome. This involves for instance, sequencing transcriptomes of multiple tissues and developmental stages at great depth and using different insert-size libraries. As a result, reference transcriptomes from closely related species are expected to be more complete and better assembled than de novo assemblies. Studies purely reference-based do not, however, allow the investigation of lineage-specific or rapidly evolving genes (i.e. when sequence divergence is too high for mapping).

A de novo assembly was generated using Oases v0.2.06 [64] on a cluster of 48 CPUs and 384 Gb of RAM. Oases is a program designed as an extension of Velvet [65], and was released as a specific tool for assembly of transcriptome sequences. Oases mitigates problems associated with the uneven coverage of contigs caused by variation in expression levels of the transcripts in the sample. A set of transcripts was generated for all odd values of K-mer ranging from 21 to 49. The obtained transcripts were merged into a single set of sequences using the default K-mer value of 27. For both the single and multiple K-mer values assembly, the average insertion length for paired-end reads was set to 170 and the minimum sequence length to 200 bp; all other parameters were left as default values. To lower the redundancy in the dataset produced by Oases, we used the clustering program CD-HIT-EST [66]. Transcripts were clustered when they shared ≥ 95% identity across ≥ 95% of the length of the smallest transcript, keeping the largest transcript in each cluster. Next, BLASTx [67] was used to align the assembled transcripts against a collection of protein databases including the datasets of five teleost fishes (fugu, medaka, stickleback and zebrafish) and mouse in the Ensembl database [68] (Ensembl Release 66, February 2012). A database of protein sequences of the family Cichlidae (NCBI RefSeq, February 2012) was also included. An E-value threshold of 10–5 was applied and only the top-BLAST match (highest E-value) was selected for each transcript. Finally, we loaded the BLASTx result in a MySql database to identify the transcripts that aligned to the same protein entries. Using MySql queries and manual inspection, one transcript per entry was retained according to its length and the alignment score to the matched protein. The obtained final set of sequences was used as reference for the read mapping and differential expression (DE) analyses.

Read mapping for each sample was carried out in CLC Genomics Workbench using the RNA-Seq module, setting the maximum number of mismatches to 2 and the minimum fraction of aligned read length to 0.9. “Broken Pairs” mapping scheme was selected. Only reads that uniquely mapped to a reference sequence were considered and the read count table was exported for the DE analyses.

Using the same parameters in CLC, read mapping for each sample was also performed using Nile Tilapia (*Oreochromis niloticus*) transcriptomic sequences as a reference. The tilapia sequences, generated by the Broad Institute (Boston, MA, USA) using Illumina next generation RNA-sequencing from skin tissues, were downloaded as de novo assembled contigs. To reduce the redundancy and retrieve a sequence set with known annotation, these contigs were processed with the same pipeline used for our de novo assembled sequences (see above).

### Differential expression analyses

Analyses of differential gene expression were carried out using the edgeR [47], DESeq [48] and baySeq [49] R packages (R v. 2.14.02). Analysis in edgeR consisted of a) excluding genes with less than 0.75 cpm from at least 7 individuals b) estimating tagwise dispersion and normalization factors c) DE was tested using the exact test, and FDR < 0.05 was considered to be evidence of DE. The analysis with DESeq was carried out using the default parameters. baySeq analysis was performed using both pairwise and multiple comparisons. The later was conducted by evaluating the posterior probabilities of genes fitting into one of the following three expression patterns: N ≠ T ≠ G, N ≠ T = G and N = T ≠ G. DEGs were annotated manually by BLASTx against nile tilapia, stickleback, zebrafish, medaka, human and mouse. Two DEGs were excluded because the assembly was considered chimerical (the alignment of the ten best BLASTx corresponded to several different peptides). The analyses methods used are listed in Table 1. Clustering analysis was performed using the package pheatmap in R (version 2.15.2) using read counts normalized by library size using the method implemented in edgeR. Read counts were further normalized for each gene’s expression intensity (median read count) to avoid expression differences becoming unrecogniseable due to outliers.

Expression levels of house-keeping genes (ACTB, RPL37a and TPT1) and genes known to affect pigmentation in vertebrates were also investigated KIT, KITLG, CSF1Ra, MC1R, SLC45a2 and PAX7. Coding sequences from tilapia, medaka or zebrafish orthologs were obtained from Ensembl (using the gene tree view) and used to identify and annotate contigs from our de novo assembly. The normalized number of reads (using the method implemented in edgeR) were inspected visually using boxplots in and tested for statistical differences using T-tests or two-sample Wilcoxon tests in R (version 2.15.2).

### Phylogenetic analyses

Identification of orthology of the DE and candidate genes was completed as follows: the Midas contig was blasted in Ensembl against medaka or zebrafish and the longest CDS was obtained. The Ensembl gene tree was used to identify paralogs and orthologs from zebra fish, medaka, stickleback, human and mouse. Tilapia CDS were retrieved from pre-Ensembl or Genbank. The Midas peptide sequence was deduced based on BLASTx results and ORF prediction of the assembled contigs. PCR primers were designed and the experimental cDNA sequences were obtained from skin cDNA libraries using standard RT-PCR and sequencing protocols (BigDye v1.1). Sequences were aligned as peptides using the muscle algorithm. Trees were constructed using maximum likelihood (PHYML) [69] with 100 bootstrap replicates in SeaView v4.3.3 [70]. Regions with non-informative gaps (those present in a single taxon) were excluded from the alignment.

## Competing interests

The authors declare that they have no competing interests.

## Authors’ contributions

AM, FH, JCJ conceived the study. All authors designed the experiments. FH, JCJ and PF conducted the molecular work (RNA extraction, library preparation), FH bred the fish. PF conducted read filtering, trimming and assembly. FH and PF performed the differential expression analysis. All authors wrote, revised and approved the final version of the manuscript. All authors read and approved the final manuscript.

## Authors’ information

Frederico Henning and Julia C Jones shared first authorship.

## Supplementary Material

Additional file 1: Table S1 Assembly and mapping statistics. Click here for file

Additional file 2: Figure S1 Number of mapped reads obtained from each group. Click here for file

Additional file 3: Figure S2 Expression levels of house-keeping genes. Click here for file

Additional file 4: Figure S3 Expression levels of known color genes. Click here for file
