# Supplementary material for: Transcriptomics of morphological color change in polychromatic Midas cichlids
Source: BMC Genomics. 2013 Mar 13;14:171. doi: 10.1186/1471-2164-14-171 (PMC3623868; doi:10.1186/1471-2164-14-171)
Supplement: Additional file 1: Table S1 — Assembly and mapping statistics. [file 1471-2164-14-171-S1.pdf]

Table S1. Assembly and mapping statistics. The table summarises the following for each sample in this study: the number of reads after demultiplexing (Raw reads); the number of reads after quality trimming, and the removal of parasite sequences (Retained reads); the reads that mapped uniquely (Uniquely mapped reads) and the reads that mapped to multiple contigs (All mapped reads) of the Midas and the Tilapia assemblies. The “All mapped reads” columns include the sum of the reads that were aligned uniquely to the assembly, and the reads that aligned to multiple locations. The retained reads were used to build the Midas *de novo* assembly. The “All mapped reads” column includes numbers smaller than those included in the “Retained reads” column because many contigs that were assembled *de novo* were excluded from the final assembly, for example: 1) contigs with low coverage; 2) contigs shorter than 200 bp; 3) contigs not annotated in the public databases (see Methods).

Sample	Raw reads	Retained reads	Reads mapped to the Midas de novo assembly		Reads mapped to the Tilapia assembly	
			Uniquely mapped reads	All mapped reads	Uniquely mapped reads	All mapped reads
N1	14731178	10186475	4957424	8155301	4824276	5075935
N2	8910388	6408408	3134179	5144105	3072004	3245589
N3	18127548	12921426	6348632	10358334	6096722	6425504
N4	4446846	3269300	1585381	2601542	1534905	1616389
N5	16858908	12367532	5999178	9781499	5573564	5904593
N6	13560288	9307106	4425962	7340742	4383714	4612665
T1	5399066	3539753	1747549	2823523	1561462	1661444
T2	16452774	11467742	5658060	9126897	5122802	5453016
T3	14206104	10209248	5001796	8245928	4937639	5196093
T4	10892948	7998658	3877919	6426558	3972563	4196976
T5	22899996	16723324	8245630	13371343	7853762	8323519
T6	21940714	16587041	8211984	13133790	7052250	7560589
G1	13250492	9753629	4806616	7824678	4554184	4812540
G2	9656418	7239759	3509752	5827557	3482279	3664884
G3	27594624	19327124	9493984	15499999	9287923	9847253
G4	19996878	15387823	7560794	12300803	7087187	7519806
G7	18349754	13750022	6698080	10806913	6112306	6491744
G8	7127242	5009844	2417269	3959352	2331612	2479916
Total	264402166	191454214	93680189	152728864	88841154	94088455
