# Supplementary figures and images for: Transcriptomics of morphological color change in polychromatic Midas cichlids
Source: BMC Genomics. 2013 Mar 13;14:171. doi: 10.1186/1471-2164-14-171 (PMC3623868; doi:10.1186/1471-2164-14-171)

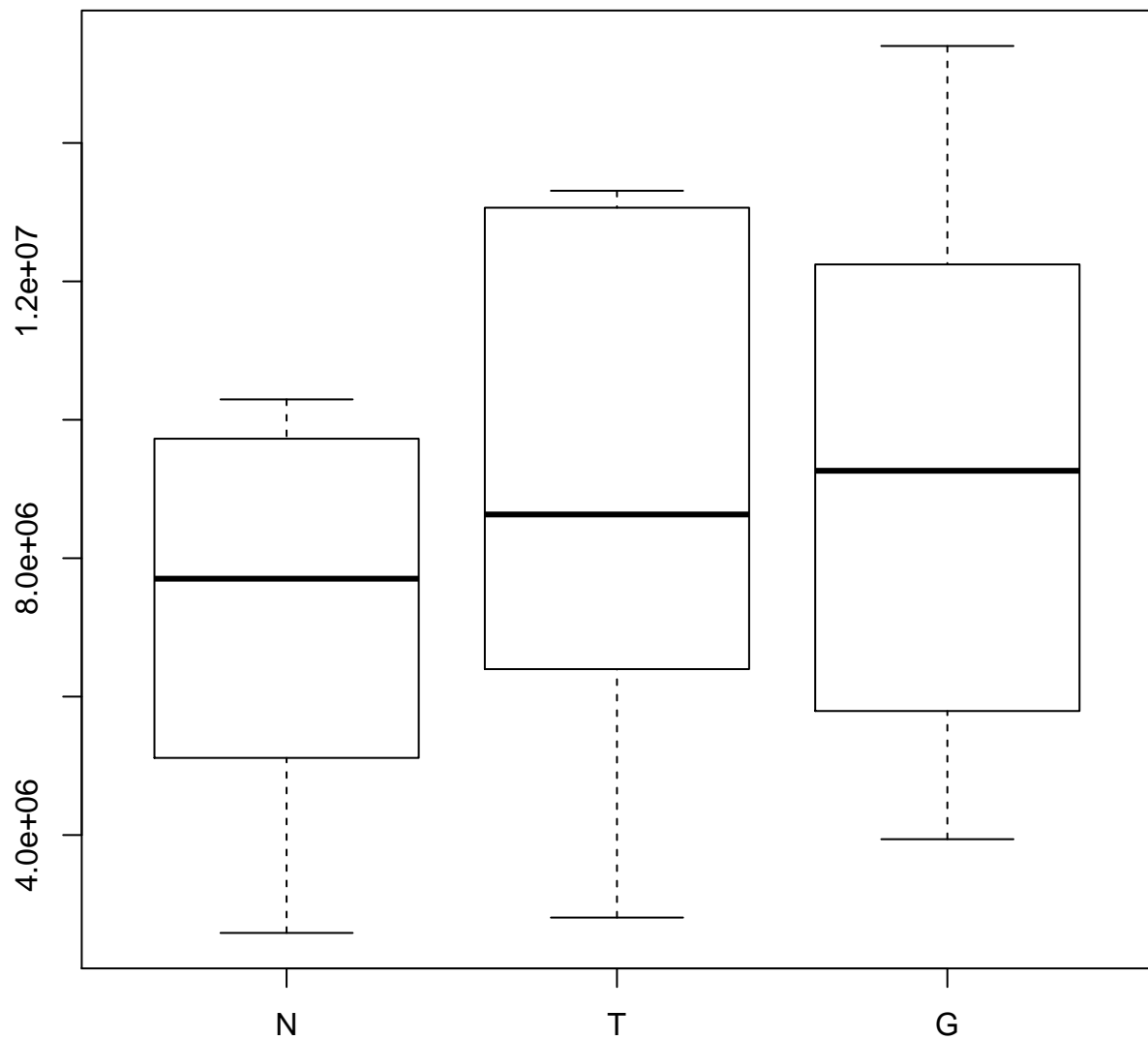

Supplement: Additional file 2: Figure S1 — Number of mapped reads obtained from each group. [file 1471-2164-14-171-S2.pdf]

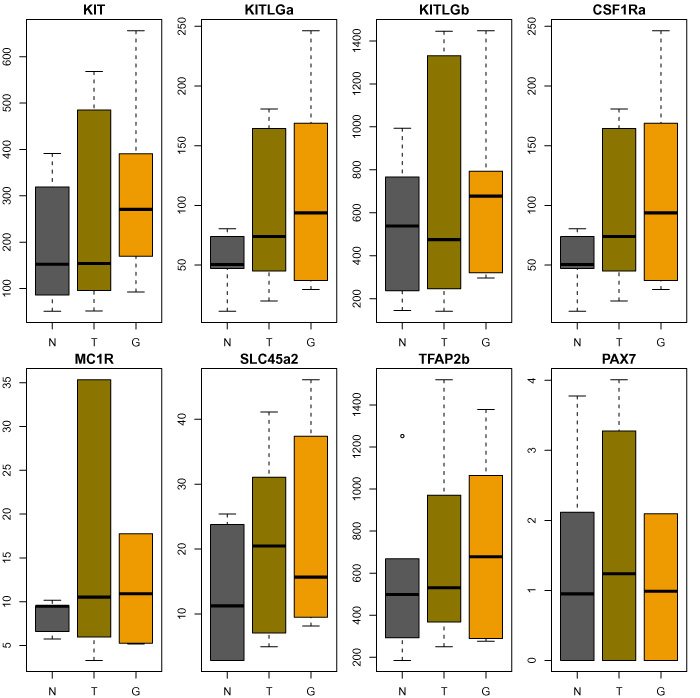

Supplement: Additional file 3: Figure S2 — Expression levels of house-keeping genes. [file 1471-2164-14-171-S3.jpeg]

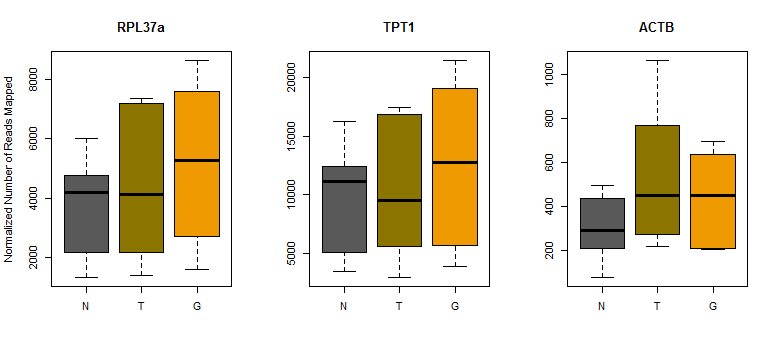

Supplement: Additional file 4: Figure S3 — Expression levels of known color genes. [file 1471-2164-14-171-S4.tiff]
